# Nutcracker syndrome: A case report and review of the literature

**DOI:** 10.3389/fsurg.2022.984500

**Published:** 2022-12-23

**Authors:** Ramandeep Kaur, David Airey

**Affiliations:** Department of Vascular Surgery, Wagga Wagga Base Hospital, Wagga Wagga, NSW, Australia

**Keywords:** nutcracker syndrome (NC), endovascular treatment, endovascular stenting, literature review, left renal vein (LRV)

## Abstract

**Background:**

Nutcracker syndrome (NS) is an uncommon condition resulting from the compression of the left renal vein (LRV) between the aorta and superior mesenteric artery (SMA), resulting in symptoms such as flank pain and hematuria.

**Case presentation:**

We present the case of a 30-year-old woman complaining of abdominal pain who was found to have nutcracker syndrome and treated with endovascular stenting of the left renal vein.

**Discussion:**

We review the literature related to endovascular treatment of NS with focus on the distribution of the sizes of stents and rates of stent migration.

**Conclusion:**

NC is a rare condition requiring a high index of suspicion for diagnosis. Endovascular treatment is a reasonable option, but its limitations must be considered.

## Introduction

Nutcracker syndrome (NS) is a rare disorder caused by extrinsic compression of the left renal vein (LRV) between the aorta and superior mesenteric artery (SMA), resulting in impaired blood outflow and congestion, causing dilation of the LRV distal to the compression (1, 2). Symptoms include gross or microscopic hematuria, flank, abdominal pain or pelvic pain, gonadal vein syndrome, varicocele, and proteinuria as well as nonspecific gastrointestinal derangements such as nausea and loss of appetite (3). It is important to distinguish NS from nutcracker phenomenon as the terms are often used interchangeably. Nutcracker phenomenon is the anatomical or radiological finding of LRV compression, while NS refers to patients who present with clinical symptoms (4).

The exact prevalence of NS is unclear because of the variability in symptoms and the absence of agreed diagnostic criteria (5, 6). Treatment options range from observation to nephrectomy depending on the degree of disease and clinician preferences. Observation is usually recommended for those who have mild hematuria or pain, and intervention should be used for patients with intractable pain, severe hematuria, renal insufficiency, and failure to respond to conservative management (1). Procedures include LRV transposition, SMA transposition, renal autotransplant, and endovascular renal vein stenting (4, 5).

## Case description

A-30-year-old woman presented to her general practitioner with episodic epigastric pain radiating to the left flank over a period of 6 months. She had a history of migraines but was otherwise well with no previous abdominal operations.

The pain was somewhat positional, improving when the left side was dependent. It was exacerbated by food—solids more than fluids—and she had noted some recent weight loss. Colonoscopy was normal; gastroscopy revealed mild gastritis, but initiation of a proton pump inhibitor (omeprazole 20 mg daily) made no improvement in symptoms. There may have also been a component of superior mesenteric artery syndrome present alongside findings consistent with nutcracker syndrome; however, this could not be substantiated as there was no imaging to support this claim. Urinalysis revealed no significant findings and no hematuria.

**Figure 1:**
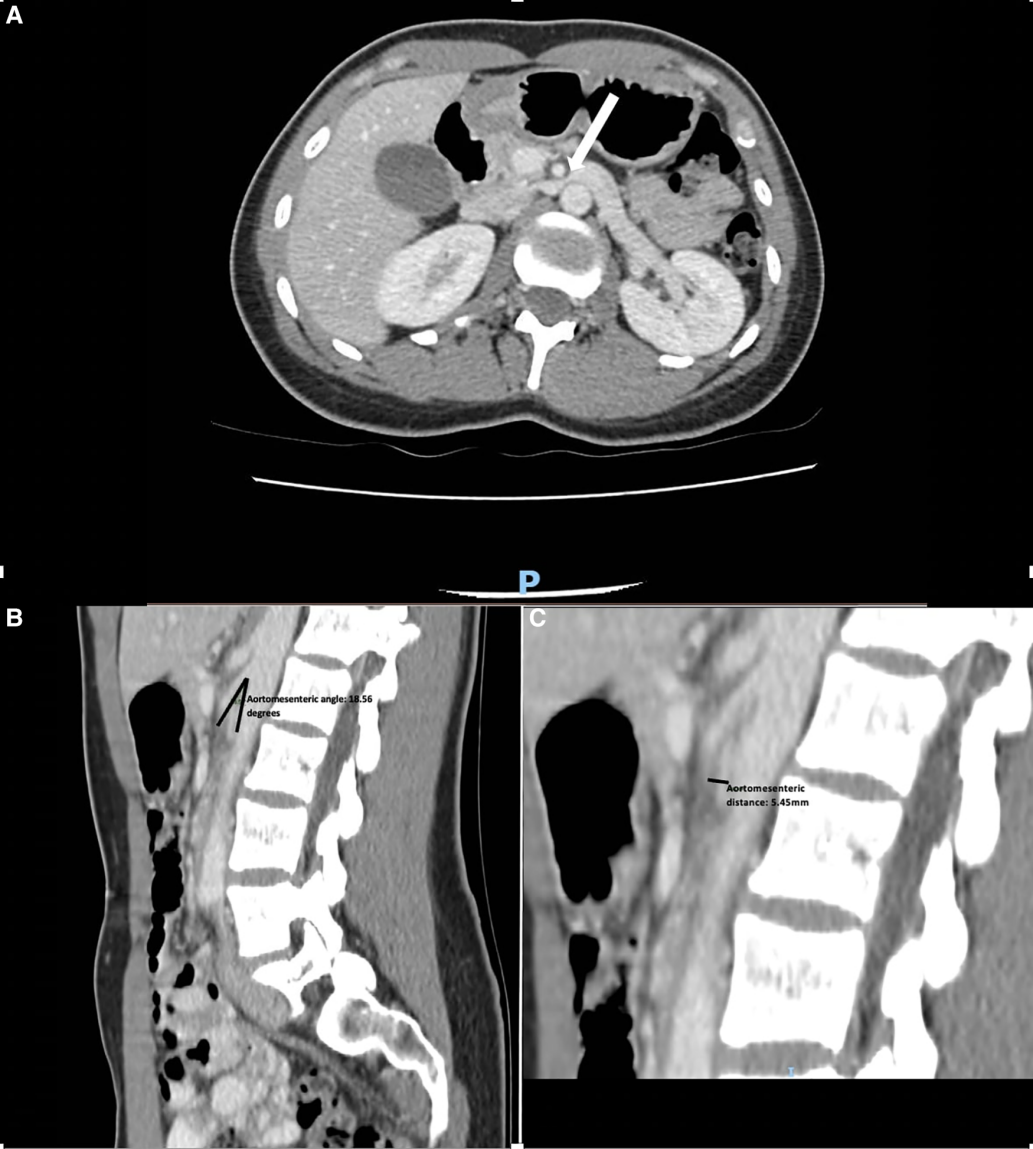
(**A**) CT scan demonstrating dilated left renal vein compressed between the superior mesenteric artery and aorta with an arrow pointing to the area of compression. (**B**) A sagittal CT scan demonstrating an aortomesenteric angle of 18.56°. (**C**) A sagittal CT scan demonstrating an aortomesenteric distance of 5.45 mm.

A CT venogram demonstrated compression of the LRV between the superior mesenteric artery and the aorta with a prominent left ovarian vein (LOV) as seen in Figure 1. Her pain worsened and became constant despite negative findings on repeat gastroscopy, and the decision was made to proceed with angiography and angioplasty with a view for intervention on the LRV.

**Figure 2:**
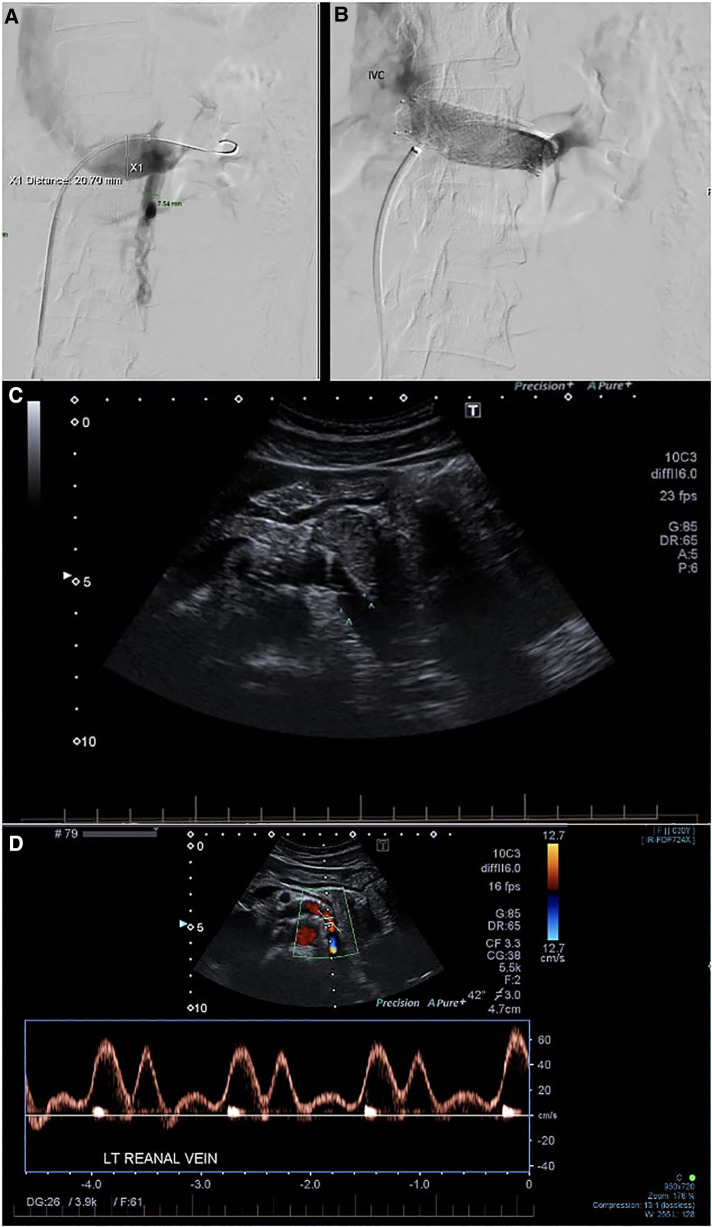
(**A**) Phlebography demonstrating stenosis of the LRV with reflux into the LOV. (**B**) Venogram after stent deployment. Reflux into the left ovarian vein is no longer seen. The distal part of the stent is somewhat constrained, but the proximal part prolapses into the IVC. (**C**) Follow-up duplex ultrasound at 3 months. (**D**) Post-procedure LRV duplex velocities and assessment of velocity ratio. IVC, inferior vena cava; LRV, left renal vein; LOV, left ovarian vein.

Access was gained *via* a 7F sheath placed in the right common femoral vein under local anesthesia. The LRV was cannulated, and angiograms were performed, demonstrating the venous dilatation and reflux into an engorged left ovarian vein (Figure 2). A 10 mm × 40 mm Amada 35 balloon (Abbot Laboratories, Chicago, IL, United States) was inflated across the point of maximal stenosis. Remarkably, the patient had instant and profound relief of symptoms, describing herself as pain-free for the first time in many months; however, once the balloon was deflated, the pain recurred over the course of 30–60 s, only to be relieved again by a second balloon inflation.

A 16 mm × 40 mm Zilver Vena self-expanding stent (Cook Medical, Bloomington, IN, United States) was deployed. No post-stent angioplasty with balloon was performed as there were already concerns regarding the oversizing of the stent. Disappointingly, the deployment was associated with an immediate recurrence of significant pain. Checking angiograms (Figure 2A) showed resolution of the stenosis and the absence of reflux into the ovarian vein. The pain improved over the course of the next few hours and was completely settled by the second postoperative day. She was discharged on 2 months of apixaban 5 mg b.i.d. and long-term aspirin 100 mg daily. She was symptom-free at 3-month follow-up with a duplex ultrasound demonstrating resolution of stenosis (Figure 2C). On further follow-up at 35 months, the patient continued to be symptom-free with no complications.

## Discussion

Management options for nutcracker syndrome range from conservative observation to endovascular stenting (EVS) or open surgery with the approach guided by the severity of symptoms and local experience (1). Endovenous intervention offers a minimally invasive approach and is becoming increasingly popular as primary management for symptomatic cases. This trend is motivated in part by promising results in the treatment of iliac vein compression such as May–Thurner syndrome, perhaps the closest clinicoanatomical analogy to NCS.

The medium-term results of EVS are promising. The six largest retrospective studies detail a total of 192 patients treated with EVS (7–12). Of these, five studies describe symptoms after the procedure as seen in Table 1. Complete or partial symptomatic improvement was reported in majority if not all patients across these studies, as demonstrated in Table 1 (7–12). Of the two patients described by Avgernios et al. (9) who had no response to treatment, one was later diagnosed with endometriosis and one remained symptomatic (and undiagnosed) despite a kidney autotransplant.

**Table 1:** Partial or complete improvement of symptoms and anticoagulant regimen according to study.

Study	Number of patients	Clinical improvement	Anticoagulation regimen
Chen et al.	61	59	Low-molecular-weight heparin 3 days followed by clopidogrel 30 days and aspirin for at least 3 months
Wang at al.	30	30	Warfarin 6–12 months
Avgernios et al.	18	15	DAPT 1–3 months then lifelong 81 mg aspirin
Li et al.	6	6	Heparin 50 U/kg infusion or subcutaneous enoxaparin followed by aspirin 100 mg for 90 days
Hartung et al.	5	5	Nadroparin 15 days and clopidogrel 6 months

DAPT, dual antiplatelet therapy.

In-stent restenosis appears to be uncommon. Avgernios et al. (9) reported three examples of restenosis requiring interventions, two of which were in patients who had previous renal vein transpositions. No other examples of in-stent restenosis and no stent fracture are recorded in these studies, although recompression associated with stent migration is described in three cases (7, 11, 13).

Postoperative selection of antithrombotic therapy is also variable, both based on choice of an agent and duration of the treatment. Dual antiplatelet therapy (DAPT) varies from 30 days to 3 months (9, 14–17). Avgernios et al. (9) commenced their patients on DAPT for 1–3 months and then on ‘baby’ aspirin (81 mg) for life. Wang et al. (8) commenced all 28 patients on warfarin therapy 6–12 months after the procedure. For our patient, we prescribed 2 months of apixaban, after which anticoagulation was ceased with no complications; however, antiplatelet therapy with 100 mg aspirin was continued.

The most feared complication of EVS is stent migration. Wu et al. (13) described stent migration in 5 out of a cohort of 75 patients (6.7%), during a mean of 55 months follow-up. Of these migrations, two stents (10 mm × 40 mm and 14 mm × 40 mm, SMART Control) moved to the right atrium and were retrieved by open cardiac surgery. One stent (10 mm × 40 mm, SMART Control) migrated to the left and was treated expectantly; one (10 mm × 14 mm, SMART Control) migrated to the right, partially prolapsing into the inferior vena cava (IVC) and was also treated expectantly,; and one (14 mm × 40 mm, Wallstent) completely prolapsed into the IVC and was removed with open surgery.

In a description of early experience of five patients, Hartung et al. (11) describe perioperative stent migration into the IVC in one case (20 mm × 60 mm, Wallstent), which required further endovascular intervention, and late migration of two stents (both 16 mm × 40 mm, Wallstent) to the right, prolapsing into the IVC and allowing recompression of the LRV and recurrence of symptoms. Chen et al. (7) (*n* = 61) described three cases of stent migration: one example of postoperative migration of a stent (10 mm × 40 mm, Wallstent) to the right atrium, requiring an open operative retrieval; one example of migration to the left (12 mm × 40 mm, Wallstent); and one example of migration to the right (10 mm × 40 mm, Wallstent). Wang et al. (8) (*n* = 30, 12–80 months follow-up), Avgernios et al. (9) (*n* = 18), and Li et al. (10) (*n* = 6) described no instances of migration.

It would seem likely that the size and type of the stent would affect the risk of migration. Of the 192 stents deployed (7–12), 146 were SMART Control, 31 were Wallstent, 15 Protégé Everflex, and 1 each of Zilver and Palmaz stents. As seen in Table 2, size data are available for 122 of the stents, with the distribution being 10 mm (*n* = 21), 12 mm (*n* = 10), 14 mm (*n* = 85), and 16 mm (*n* = 6). Hartung et al. (11) deployed a 20-mm diameter Wallstent, but this was later retrieved after migration. For stents that migrated, the size distribution was 10 mm (*n* = 4), 12 mm (*n* = 1), 14 mm (*n* = 2), 16 mm (*n* = 2), and 20 mm (*n* = 1). Of the stents subject to migration, six were Wallstent and four were SMART Control stents.

**Table 2:** Distribution of stent migration according to stent size.

Size of stent (mm)	Number of stents (*n*)	Number migrated (*n*)	Percentage
10	21	4	19.0
12	10	1	10.0
14	85	2	2.4
16	6	2	33.3
20	1	1	100

The left renal vein diameter typically measures 12.0 ± 2.0 mm in cadaveric studies (16–19) but is known to expand during the Valsalva maneuver (20). The distal portion is commonly dilated in the presence of outflow obstruction. With our patient, the distal LRV measured up to 12 mm on the preoperative CT scan but appeared larger on the catheter venograms (Figure 2). To aid with LRV stent sizing accuracy, intravascular ultrasound (IVUS) is becoming an increasingly common mode of imaging utilized during angiography. It can aid with the sizing of the LRV stent and has the potential to reduce the risk of oversizing or undersizing the stent (12). However, further evidence is required to support the benefits of IVUS in LRV stenting.

The stent chosen was a 16 mm × 60 mm Zilver Vena stent with the primary concern of preventing migration. The Zilver Vena stent is a dedicated venous stent and although limited experience in the renal vein is described, it has shown good safety and efficacy in iliocaval use (21–23) It was disappointing that the deployment of the stent was associated with a recurrence of pain that had been entirely absent with simple balloon inflation. We interpreted this as caused by the intrinsic stretch of the LRV by the stent. Fortunately, the pain subsided after the procedure and had completely resolved by the second postoperative day.

## Conclusion

NCS is a rare condition requiring a high index of suspicion for diagnosis. Treatment options vary based on severity; however, considering EVS is becoming an increasingly common and minimally invasive solution, it seems particularly efficacious for the treatment of venous stenosis or compression which contributes to flank pain in such cases. In this case, EVS has demonstrated effective results at follow-up with no long-term consequences. The sizing of stents remains a matter for judgment, and intravascular ultrasound could be considered to aid planning. Although larger stents may reduce the risk of migration, oversizing is not without consequences. Further data will assist development of optimal treatment algorithms.

## Data Availability

The original contributions presented in the study are included in the article/Supplementary Material, further inquiries can be directed to the corresponding author.
